# Supplementary material for: Epidemiological Aspects of Maternal and Congenital Toxoplasmosis in Panama
Source: Pathogens. 2021 Jun 17;10(6):764. doi: 10.3390/pathogens10060764 (PMC8234371; doi:10.3390/pathogens10060764)
Supplement: Supplementary file 1 [file pathogens-10-00764-s001.zip › pathogens-1238369-supplementary.pdf]

Table S1
Regions and communities surveyed in this study.

Region	Communities
Metro	San Francisco, Bethania, Río Abajo, Parque Lefevre, Juan Díaz.
West	Barrio Balboa, Barrio Colón, El Coco, Guadalupe, Playa Leona, Puerto caimito, Arraiján, Burunga, Cerro Silvestre, Juan Demóstenes Arosemena, Nuevo Emperador, Veracruz, Vista Alegre, Chame, Capira.
Central	Curundu, Ancon, Santa Ana, Calidonia, Bella Vista, El Chorrillo.
North	Alcalde Díaz, Chilibre, Las Cumbres, Ernesto Córdoba Campos.
East	Las Mañanitas, Pacora, Tocumen, 24 de Diciembre, Pedregal, Chepo.
San Miguelito	Mateo Iturralde, José Domingo Espinar, Victoriano Lorenzo, Amelia Denis de Icaza, Arnulfo Arias, Belisario Porras, Belisario Frias, Omar Torrijos, Rufina Alfaro.
